# A Comparison of Subjective Symptoms of Two Types of Silicone Hydrogel One-Day Disposable Contact Lenses

**DOI:** 10.7759/cureus.71553

**Published:** 2024-10-15

**Authors:** Tatsuya Mimura, Hidetaka Noma, Masao Yamaguchi, Koichiro Shinbo

**Affiliations:** 1 Ophthalmology, Teikyo University School of Medicine, Tokyo, JPN; 2 Department of Ophthalmology, Tokyo Medical University Ibaraki Medical Center, Inashiki-gun, JPN; 3 Ophthalmology, Nerima Station West Eye Clinic, Tokyo, JPN

**Keywords:** daily disposable soft contact lens, narafilcon a, questionnaire survey, silicone hydrogel, verofilcon a

## Abstract

Purpose: This study aimed to evaluate the comfort and discomfort of two types of soft contact lenses (SCLs) by comparing their quality of vision, fit, subjective dryness, and handling.

Methods: A total of 62 SCL wearers with contact lens (CL) discomfort were recruited. They were fitted with narafilcon A and verofilcon A for two weeks each by crossover. The comfort of the two types of SCLs was compared by having each participant answer a modified 5-Item Dry Eye Questionnaire (m-DEQ5, scores for vision, wearing comfort, and handling: the total score of the five items is a maximum of 25, higher being better) and modified Japanese version of the 8-item Contact Lens Dry Eye Questionnaire (m-J-CLDEQ-8: scores for discomfort and dry eye symptoms: the total score of the eight items is a maximum of 44; lower is better) survey forms.

Results*:* Overall, the m-DEQ5 and m-J-CLDEQ-8 scores for the two SCLs of narafilcon A and verofilcon A were higher than the median of 3.0 points. Scores for 4 of 5 items and total scores in m-DEQ5 (18.7 ± 3.4 vs. 21.6 ± 2.5) were significantly higher and better in verofilcon A than in narafilcon A (p<0.01). Scores for 8 of 8 items and total scores on the m-J-CLDEQ-8 (15.2 ± 7.9 vs. 8.4 ± 4.9) were also significantly lower and better in verofilcon A than in narafilcon A (p<0.05).

Conclusion: Verofilcon A provided excellent vision, comfort, and ease of handling and was less likely to cause dry eye symptoms or eye discomfort. These results suggest that verofilcon A is an excellent option for patients with dry eye symptoms and CL discomfort.

## Introduction

Over the past two decades, soft contact lens (SCL) materials have significantly changed. In particular, the introduction of silicone hydrogel (SiHy) materials has dramatically improved the fit of SCLs. SiHy-SCLs combine conventional hydrogel materials with highly gas-permeable silicone materials while maintaining transparency for clinical applications [1]. These hydrogels contain a silicone polymer with high gas permeability and adequate oxygen permeability, even when water content is low [1]. Therefore, SiHy-SCLs exhibit features such as less dryness, less staining, and less hyperemia.

However, eye discomfort and dryness are important problems associated with SCL wear, making wearing the lens difficult for patients with dry eye symptoms [2]. Hence, improved surface wettability of SCL remains an important aspect of SCL biocompatibility. Two types of factors can cause CL discomfort (CLD): CL-related and environment-related. CL-related factors include (1) material (lubricity and water content), (2) design (edge, base curve, asphericity), (3) wearing comfort, (4) wearing time and interval between wear, and (5) SCL care system (chemical composition, regimen) [3].

The prevalence of CLD is estimated to be 23-94% in patients with symptoms attributable to CLs [2-6]. In a Canadian questionnaire-based epidemiological study of dry eye (CANDEES), 50.1% of 3,285 SCL wearers had dry eye symptoms compared to 21.7% of non-SCL wearers [7]. The dropout rate of SCL wearers within four months of wearing SCL was estimated to be approximately 40% [2]. Another study reported that 50-75% of CL wearers experience CL discomfort, and 12 -51% stop using CL [2,3]. In a U.S.-based study, 564 (52%) of 1,092 SCL wearers were aware of discomfort and dryness [8]. This included 23% dryness, 13% discomfort, and 27% uncomfortable wearing for at least two hours [8]. Therefore, patients who are inherently or occupationally prone to CLD should be advised to use SCLs and lens-care systems that are more comfortable for their eyes.

Verofilcon A daily disposable hydrophilic polymer-layered CLs (PRECISION 1®, Alcon Japan Ltd., Tokyo, Japan) are unique with the characteristics of SiHy lenses. These aqueous-gradient lenses have a SiHy core that provides high oxygen transmission and a high tensile modulus. This proprietary technology is called "SMART SURFACE™ Technology" and has been used in the manufacturing process of verofilcon A and delefilcon A [9,10]. Unlike SiHy lenses, verofilcon A covers the SCL surface with a hydrophilic polymer with high water retention properties, resulting in very high water content (51% in the core) and over 80% water content on the SCL surface [9,10]. Thus, verofilcon A is comfortable to wear and easy to handle [11,12]. The purpose of this clinical study was to evaluate the satisfaction and tolerability of verofilcon A hydrophilic polymer-layered daily disposable SCLs.

## Materials and methods

Research design

This was an investigator-initiated, prospective, randomized, crossover, two-arm trial. The study was conducted in accordance with the ethical guidelines of the Declaration of Helsinki (World Medical Association 2013) and the Ethical Guidelines for Medical Health. The trial involving human participants was approved by the Teikyo University Ethical Review Committee (#19-211). A series of studies, including this one, have been registered as clinical trials in the University Medical Information Network for Clinical Trials (UMIN-CTR; UMIN registration numbers: UMIN000041107. The research project began on April 1, 2021, and participants were recruited between March 1, 2022, and July 31, 2022.

This study was undertaken between April 2021 and July 2022 at the outpatient clinic of Nerima Station West Eye Clinic and the Department of Ophthalmology, Teikyo University School of Medicine. Written informed consent was obtained from all the participants after a complete explanation of the study content.

Participants

The inclusion criteria for the study required participants to be in good health, aged 12 years or older, and to have myopic astigmatism. Their refractive error needed to fall within the range of -0.5 diopters (D) to -6.0D, and they had to demonstrate a best corrected visual acuity of 20/25 or better. Additionally, participants were required to exhibit symptoms of dry eye and CL discomfort (CLD).

On the other hand, the exclusion criteria specified that individuals younger than 12 years of age, those with ocular or systemic diseases, individuals with a history of refractive surgery, and those with corneal epithelial erosion were not eligible for the study. Artificial tear drops were allowed on the CLs during the study. Participants were selected from patients who used disposable SCLs daily.

**Figure 1:**
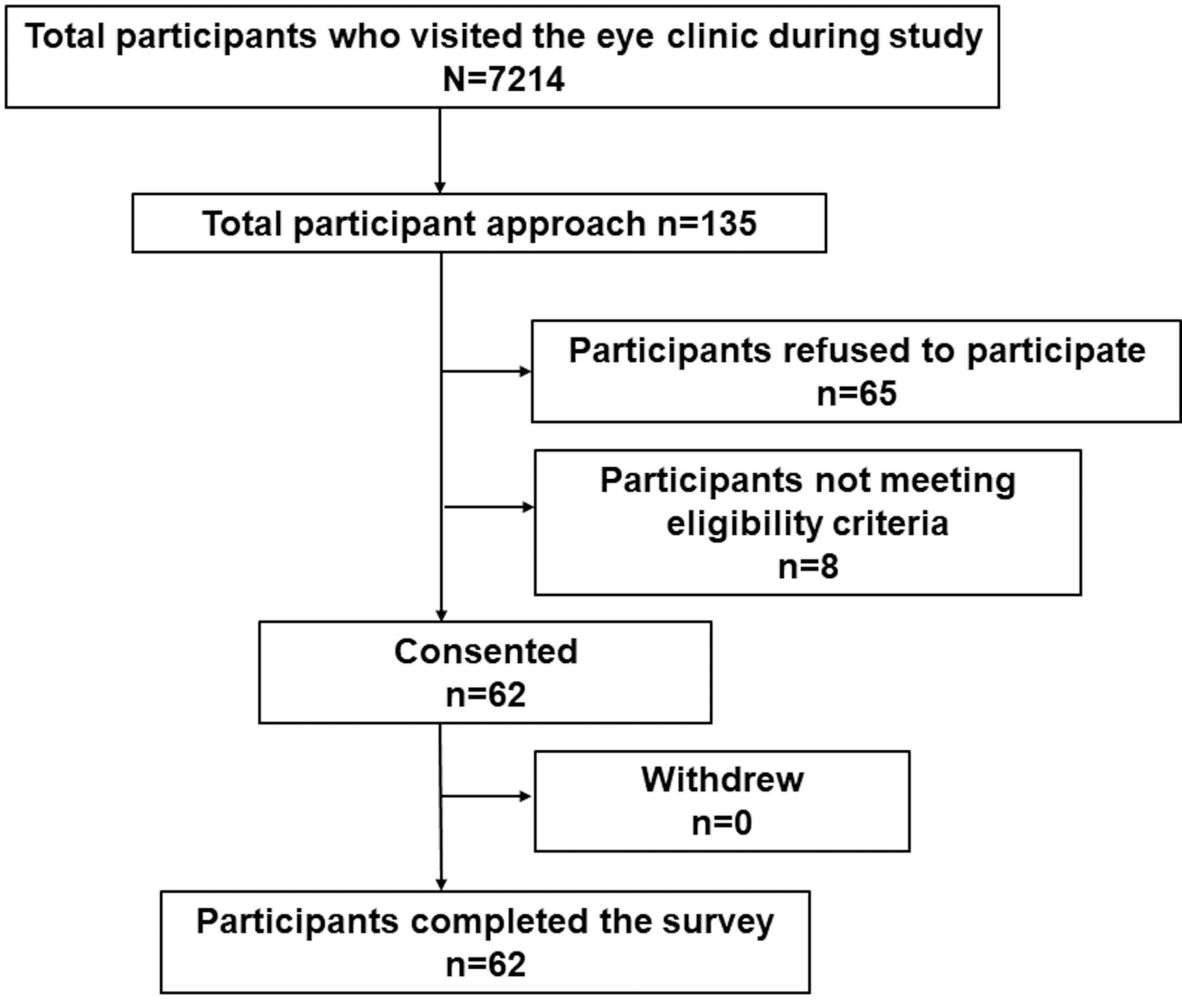
The CONSORT flow diagram showing participants recruitment process. CONSORT=Consolidated Standards of Reporting Trials

Figure 1 shows the Consolidated Standards of Reporting Trials (CONSORT) diagram to illustrate the recruitment process of participants. The study was designed to analyze data from more than 50 individuals, and 62 individuals were ultimately enrolled in the study. In all, a total of 62 participants were included in this study. The age of the included patients ranged from 14 to 39 years (mean ± deviation, 26.3 ± 8.4). A total of 21 males and 41 females participated.

Study schedule

**Figure 2:**
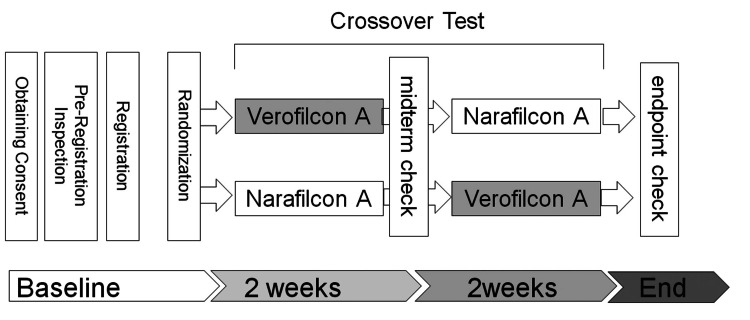
Schematic overview of the crossover study design and the experimental protocol.

A crossover study was conducted to compare the comfort of wearing narafilcon A (1-Day Acuvue® True Eye) and verofilcon A (PRECISION1) (Figure 2). The first lens was randomly selected, used for two weeks and then switched to the other lens. 　

Randomization for SCL selection was performed using the randomization and trial supply management (RTSM) of Veiedoc Admin® (Veiedoc Japan Corporation, Tokyo, Japan) with dynamic allocation by the Pocock-Simon method. One of the two SCLs selected by the system was used as the first lens. A questionnaire-based survey was conducted on the use of the two types of SCLs by each subject. Examinations were conducted at baseline and after two and four weeks. Participants were asked to complete questionnaires about wearing SCLs at two and four weeks after the start of the study. A total of 62 participants were enrolled, and all participants completed the one-month follow-up study.

Characteristics of the evaluated SCLs

Narafilcon A and verofilcon A disposable SiHy SCLs were used daily. The characteristics of the two SCL types are listed in Table 1. Narafilcon A is a SiHy material introduced in 2009 and available as Johnson & Johnson's daily disposable ACUVUE® lens 1-Day ACUVUE® TruEye. Narafilcon A has a high water content of 46% and high oxygen permeability (Dk; 100 × 10-11 barriers) [9], making it a widely used lens. Verofilcon A, on the other hand, is made from a new high oxygen permeability (Dk; 90 × 10−11 barriers) material with a 2-3 µm thick water surface that has over 80% water content at the surface and is a Class 1 ultraviolet blocker (≥ 90% of UVA, ≥ 99% of UVB) [9]. Verofilcon A SCLs have a smooth surface owing to the SMART SURFACE® technology.

**Table 1:** Characteristics of the soft contact lenses (SCLs). CT=center thickness of contact lens; FDA=Food and Drug Administration; USAN=United States adopted names; HEMA=2-Hydroxyethyl methacrylate; mPDMS=mono-methacrylate poly dimethylsiloxane; DMA=NN'-dimethyl acrylamide; GPDMS=Glycerol-functionalized polydimethylsiloxane; NVP=N-vinyl pyrrolidone.

Property	Narafilcon A	Verofilcon A
Water Content (%)	46	Core 51 / Surface 80
Oxygen Permeability (Dk)	100	90
Oxygen Transmissibility (Dk/L)	118	100
Diameter (mm)	14.2	14.2
Base Curve (mm)	9.0/8.5	8.7/8.3
CT (mm)	0.085	0.09
Color	Blue	Light blue
Surface (Ionic/Non-ionic)	Non-ionic	Non-ionic
FDA group	I	II
USAN Nomenclature	Narafilcon A	Verofilcon A
Principal Components	2-HEMA，mPDMS，DMA	mPDMS， GPDMS， NVP

Questionnaire

Two CL-wear questionnaires were administered. The first was a modified 5-Item Dry Eye Questionnaire (m-DEQ5) used by Grant et al. on SCL wearing comfort [11]. The second was on discomfort during SCL wear, modified from the Japanese version of the 8-item Contact Lens Dry Eye Questionnaire (m-J-CLDEQ-8) [13-15].

Questionnaires were administered two and four weeks after wearing the SCLs. The comfort and discomfort levels of the SCL were analyzed based on the scores from each response. The total score on Questionnaire 1 was used as the total comfort score (maximum value of 25), and the total score on Questionnaire 2 was used as the total discomfort score (maximum value of 44).

Statistical analyses

Two-tailed paired Student’s t-test or Wilcoxon signed-rank test was used to determine the significance of differences between the two groups. Data are expressed as means ± standard deviation or percentage. Statistical analyses were performed using SAS System software version 9.1 (SAS Institute Inc., Cary, NC, USA), and significance was set at p <0.05.

## Results

Subject characteristics

The demographic and baseline characteristics of the participants are presented in Table 1. A total of 62 participants were enrolled in this study. All 62 participants completed the study, and none dropped out. The right and left eyes of subjects had a similar mean refractive error (-4.2 ± 1.9 D vs. -4.1 ± 1.7 D), mean sphere (-3.9 ± 1.9 D vs. -3.7 ± 1.6 D), and mean cylinder (-0.7 ± 0.4 D vs. -0.7 ± 0.4 D).

**Table 2:** Demographics and baseline characteristics of the study participants. SD=standard deviation, *refraction (D)=sphere + cylinder/2.

Parameters	Observed Values
Age (years)	
Mean ± SD	26.3 ± 8.4
Range: minimum, maximum	14 – 39
Sex, n (%)	
Females	41 (66.1%)
Males	21 (33.9%)
Baseline refraction, mean ± SD, diopters (D)	
*Refractive error, right eye	-4.2 ± 1.9
*Refraction error, left eye	-4.1 ± 1.7
Sphere, right eye	-3.9 ± 1.9
Sphere, left eye	-3.7 ± 1.6
Cylinder, right eye	-0.7 ± 0.4
Cylinder, left eye	-0.7 ± 0.4
Contact lens power, right eye	-3.3 ± 1.5
Contact lens power, left eye	-3.1 ± 1.5
Baseline keratometry, mean (mm)	
Right eye K1 / right eye K2	7.8/7.6
Left eye K1 / left eye K2	7.87.6

A. Comfort level on SCL wear (Questionnaire 1)

**Figure 3:**
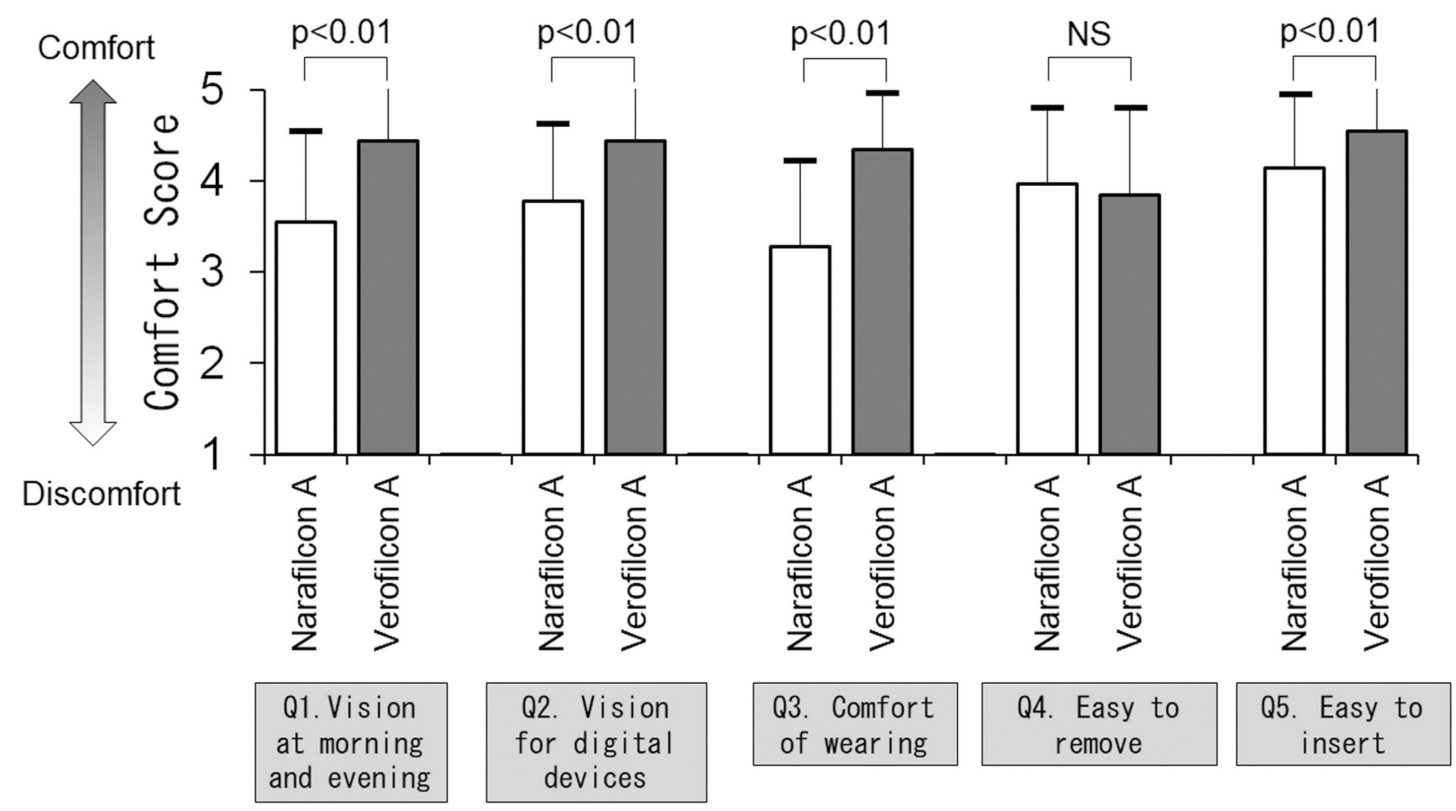
Comfort level on soft contact lens (SCL) wear (Questionnaire 1). The comfort level of participants wearing SCL (n=62) is compared between narafilcon A and verofilcon A (Wilcoxon signed-rank test). NS=not significant.

The comfort levels of the participants wearing SCL are shown in Figure 3. Both narafilcon A and verofilcon A had better mean comfort scores than the median of the three in both groups. Verofilcon A scored better mean comfort scores than narafilcon A on four of the five comfort score questions - Q1: “Provide clear vision at the beginning and end of the day” (3.5 ± 1.0 vs. 4.4 ± 0.6, p<0.01); Q2: “Provide clear vision when viewing digital devices such as mobile phones and computer screens” (3.8 ± 0.9 vs. 4.4 ± 0.6, p<0.01); Q3: “Allows me to comfortably wear SCLs all day long” (3.3 ± 0.9 vs. 4.3 ± 0.6, p<0.01); and Q5: “Easy to place SCLs on my eye” (4.1 ± 0.8 vs. 4.5 ± 0.7, p<0.01). On the other hand, the response to Q4, " Easy to remove SCLs at the end of the day," was not significantly different between the two groups (4.0 ± 0.8 vs. 3.8 ± 1.0, p=0.37).

B. Discomfort level on SCL wear (Questionnaire 2)

**Figure 4:**
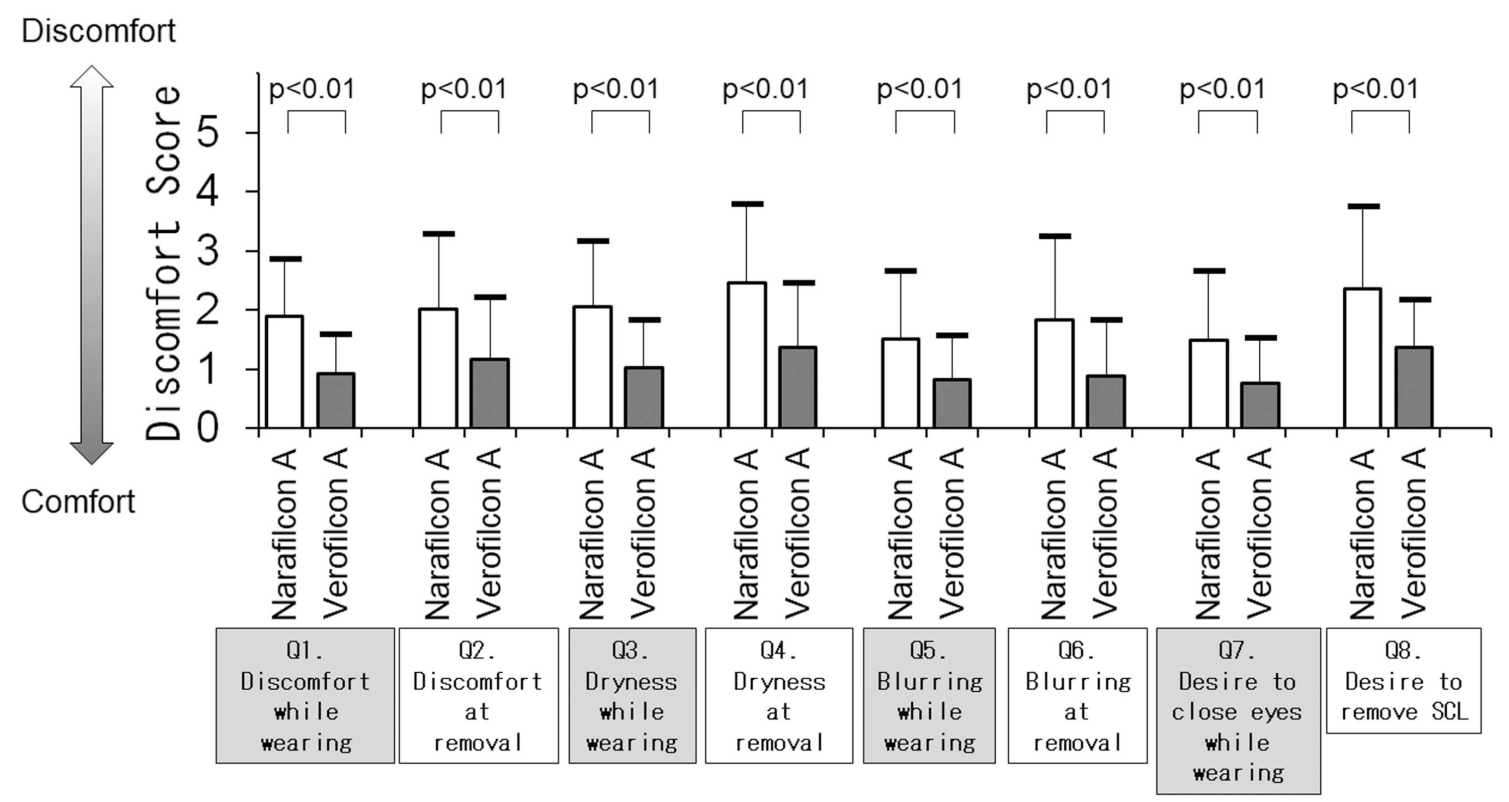
Discomfort level on soft contact lens (SCL) wear (Questionnaire 2). The discomfort level of participants wearing SCL is compared between narafilcon A and verofilcon A (Wilcoxon signed-rank test).

Discomfort levels related to wearing SCL are shown in Figure 4. For both narafilcon A and verofilcon A, the mean of all discomfort scores was lower than the median of 2 or 3 for both groups (no discomfort). Verofilcon A scored lower mean discomfort scores (more comfortable) than narafilcon A on all eight discomfort scores: Q1 “discomfort during the day while wearing SCL” (1.9 ± 1.0 vs. 0.9 ± 0.6, p<0.01), Q2 “discomfort just before removing SCL” (2.3 ± 1.4 vs. 1.2 ± 1.0, p<0.01), Q3 “dryness during the day while wearing SCL” (2.1 ± 1.1 vs. 1.0 ± 0.8, p<0.01), Q4 “dryness just before removing SCL” (2.5 ± 1.3 vs. 1.4 ± 1.1 1.1, p<0.01), Q5 “blurring during the day while wearing SCL” (1.5 ± 1.1 vs. 0.8 ± 0.7, p<0.01), Q6 “blurring just before removing SCL” (1.8 ± 1.4 vs. 0.9 ± 0.9, p<0.01), Q7 “want to close eyes while wearing SCL” (1.5 ± 1.2 vs. 0.8 ± 0.7, p<0.01), Q8 “want to remove it while wearing SCL” (2.4 ± 1.4 vs. 1.4 ± 0.8, p<0.01).

Total scores for comfort and discomfort levels

**Figure 5:**
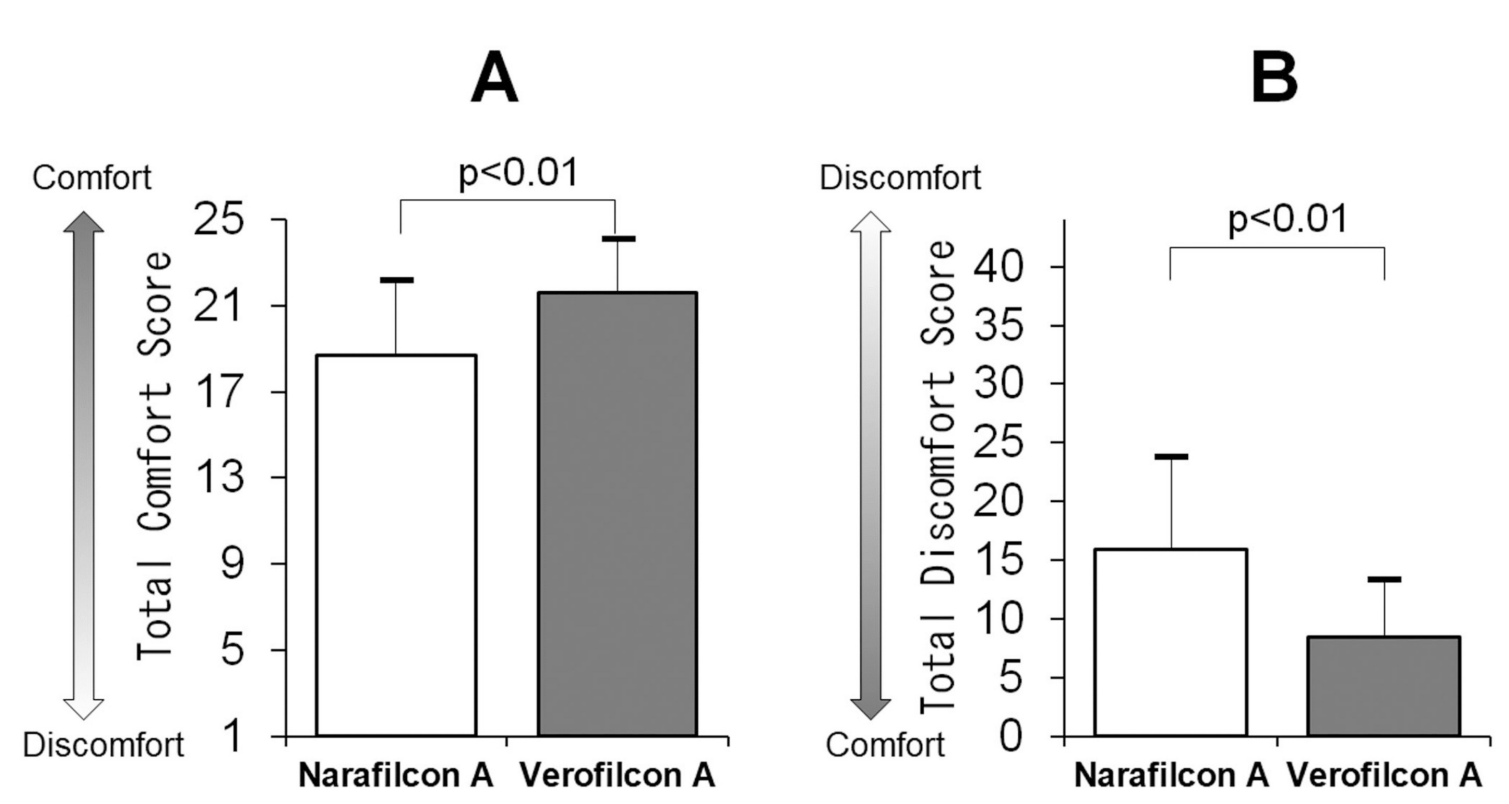
Total scores for comfort level and discomfort level. Total scores for comfort level (A: maximum value of 25) and discomfort level (B: maximum value of 44). A. Comfort level on soft contact lens (SCL) wear (Questionnaire 1). B. Discomfort level on SCL wear (Questionnaire 2). Both scores are compared between narafilcon A and verofilcon A (Wilcoxon signed-rank test).

The total scores for comfort and discomfort levels are depicted in Figure 5. As seen from the figure, the total comfort score was higher for verofilcon A than narafilcon A (18.7 ± 3.4 vs. 21.6 ± 2.5, p<0.01, Figure 5A). In contrast, the total discomfort score was significantly lower for verofilcon A than narafilcon A (15.9 ± 7.8 vs. 8.4 ± 4.9, p<0.01, Figure 5B).

## Discussion

Based on the responses to four of the satisfaction questionnaires, our results showed that verofilcon A was more comfortable than narafilcon A. On the other hand, verofilcon A was less uncomfortable than narafilcon A for all nine items included in the discomfort questionnaire. This indicates that verofilcon A is a comfortable fit for patients with dry eye symptoms.

In terms of adverse events, both types of lenses were used comfortably for one month in all 62 patients without any difficulties. Furthermore, none of the patients reported any problems while wearing SCLs. This suggests that both SCLs can be used continuously without any defects.

All comfort scores were better than the median of the three points for both SCLs. Verofilcon A was more comfortable for four of the five comfort-related questions. Narafilcon A has traditionally been used with high wearing comfort and has attracted many SCL users for a long time [16,17]. For example, one study randomly assigned 74 subjects with no previous CL experience to wear narafilcon A lenses or no CLs for 12 months [17]. Comfort scores and inflammation, such as hyperemia, after a year of wear, were similar in the narafilcon A and SCL-naïve groups. There was also a clear improvement in comfort during the first month of wearing narafilcon A [17]. This study showed that narafilcon A provides excellent and comfortable corrected vision and minimizes changes in the physiological function of the eye. With its high comfort level, narafilcon A has been a widely used lens over the past decade.

Sarac et al. also evaluated the tear fluid osmolarity and ocular comfort in 15 first-time SCL wearers at four and eight hours after wearing narafilcon A. The mean baseline, four- and eight-hour tear fluid osmolarity values were 294 ± 13.65, 300.9 ± 11.3, and 298.80 ± 7.2 mOsm/L, respectively. The mean comfort score decreased significantly from 9.80 ± 0.45 at four hours to 7.80 ± 0.84 at eight hours. However, the increase in tear osmolarity was below the dry eye cut-off and was not associated with eye comfort. In other words, tear fluid osmolarity did not increase after narafilcon A wear, indicating that dry eye is not associated with worsening eye comfort with narafilcon A [16]. Thus, verofilcon A has a better comfort level than narafilcon A, suggesting that verofilcon A is more comfortable for patients with dry eye symptoms.

Discomfort and dry eyes are the most significant factors that make SCLs unsatisfactory to wear [18-20]. Discomfort and dry eye symptoms that occur during SCL wear contribute to the discontinuation of SCL wear. In an online Facebook survey of 4207 people in Canada from 2008 to 2010, an alarming 40% of patients abandoned SCL wearing within four months [21]. Compared to patients who discontinued SCLs, those who continued wearing their SCLs wore more SiHy SCLs (49% vs. 38%). The authors reported that the main reason for abandoning SCL wear was discomfort (24%), followed by dryness (20%), hyperemia (7%), and cost (7%) [20]. In another study, 110 subjects (aged 13-19 years) with no previous CL experience were randomly assigned to nelfilcon A (Dailies AquaComfort Plus) SCL or glasses for six months. By the sixth month of the study period, 13 of the 110 patients had discontinued the study. This included 10 (17.5%) in the SCL group and three (5.7%) in the eyeglasses group, with the proportion of subjects who discontinued being significantly higher in the SCL group than in the eyeglasses group (p = 0.04) [22]. Although our study had a shorter duration (one month) than other studies, none of the subjects abandoned wearing SCLs during the study period. This may be because of the comfort of wearing narafilcon A and verofilcon A.

Another survey was conducted in New Zealand and Australia on the use of verofilcon A, which we used in our study [11]. The study included 218 patients who were already using daily disposable SCLs and had switched from other SCLs to verofilcon A, a SiHy SCL, and 129 first-time CL wearers who used verofilcon A. Of the 129 first-time CL wearers, 75% agreed that the SLCs were comfortable to wear throughout the day, 78% agreed that the SCLs provided as clear a view at the end of the day as at the beginning, and 79% agreed that wearing the SCLs provided a clear vision of text and photos on digital devices [11]. This study and our results show that verofilcon A can provide a clear vision at all times for SCL wearers.

However, the percentage of participants who responded "Yes" to "Easy to remove SCLs at the end of the day" did not differ between the narafilcon A and verofilcon A groups. The better the fit and adhesion of the SCLs to the cornea, the more difficult it is, theoretically, to remove them. While verofilcon A was more comfortable to wear than narafilcon A, the ease of SCL removal was similar in both groups. In addition, because participants were recruited from among patients with dry eye symptoms, dry eye symptoms may have affected SCL removal but not the ease of its removal.

The results of the questionnaire on discomfort in Questionnaire 2 showed that verofilcon A was less uncomfortable than narafilcon A in all eight questions. As mentioned in the first half of the discussion, narafilcon A has long been used as a reputable lens by a wide range of SCL users [16,17]. The results showed that verofilcon A is less uncomfortable in patients with dry eye symptoms than narafilcon A, making verofilcon A a promising lens for SCL users who have traditionally used them despite dry eye problems. In other words, the findings indicate that switching from conventional SCLs to verofilcon A may lead to less discomfort, permitting a more comfortable life for CLs.

Why was the discomfort level lower with verofilcon A than with narafilcon A? Dry eyes are a major cause of SCL dissatisfaction and a major reason for reduced or abandoned SCL use [23]. In the final report of a study of 4207 cases, approximately 23% of those surveyed had permanently stopped wearing CLs, with persistent discomfort and dryness being the main reasons for dropout [21]. Dropout rates are reportedly lower among SiHy wearers [21]. In other words, a better way to reduce SCL dropout is to make it easier to wear and alleviate dry eye symptoms.

SiHy combines two materials: a hydrophobic silicone with excellent oxygen permeability and a hydrophilic polymer. It has high oxygen permeability and generally low water content [24]. On the other hand, verofilcon A is a lens with extremely high oxygen permeability (90 Dk) and oxygen transmission (100 Dk/L), which are characteristics of SiHy and overcome the disadvantages of SiHy’s low water content (high water content; center of SCL: 51% water content; near SCL surface: 80% or more). These special features of verofilcon A may increase the adhesion and moisture retention of the SCL to the ocular surface and enhance the satisfaction of SCL wearers.

This study has several limitations. First, this was a randomized, two-arm study that compared only two types of SCLs. A number of parameters are related to the comfort of wearing CLs, and future comparisons with other lenses will likely provide new information about the comfort level of SCLs. Second, the study period was one month, and a long-term comparative study is needed. Third, this was primarily a questionnaire survey of the subjects, and additional research on other findings, such as tear fluid dynamics and lens movement during verofilcon A wear, would be useful.

## Conclusions

In conclusion, the comfort and quality of vision with verofilcon A were satisfactory in SCL wearers with dry eye symptoms and CLD. The comfort of verofilcon A may provide new opportunities for those who have given up wearing SCLs due to dry eye symptoms.

## Appendices

**Table 3:** Questionnaire about eye comfort: Questionnaire 1. Modified scores used by Grant et al. [10]. SCL = soft contact lens.

Please answer the following questions on a scale of 1-5. 1=Strongly disagree, 2=Disagree, 3=Undecided, 4=Agree, 5=Strongly agree.					
Q1 Provide clear vision at the beginning and end of the day.	1	2	3	4	5
Q2 Provide clear vision when viewing digital devices such as mobile phone and computer screen.	1	2	3	4	5
Q3 Allows me to comfortably wear SCLs all day long.	1	2	3	4	5
Q4 Easy to remove SCLs at the end of the day.	1	2	3	4	5
Q5 Easy to place SCLs on my eye.	1	2	3	4	5

**Table 4:** Questionnaire about eye discomfort: Questionnaire 2. Modified scores used by Chalmers et al. [12].  SCL = soft contact lens.

Please choose the best answer to the following question.	
Q1 Discomfort during the day while wearing SCL	0=Never
	1=Rarely
	2=Sometimes
	3=Frequently
	4=Constantly
Q2 Discomfort just before removing SCL	0=Never
	1=Not at all
	3=Somewhat Intense
	3=Somewhat Intense
	4=Intense
	5=Very intense
Q3 Dryness during the day while wearing SCL	0=Never
	1=Rarely
	2=Sometimes
	3=Frequently
	4=Constantly
Q4 Dryness just before removing SCL	0=Never
	1=Not at all
	2=Slightly Intense
	3=Somewhat Intense
	4=Intense
	5=Very intense
Q5 Blurring during the day while wearing SCL	0=Never
	1=Rarely
	2=Sometimes
	3=Frequently
	4=Constantly
Q6 Blurring just before removing SCL	0=Never
	1=Not at all
	2=Slightly Intense
	3=Somewhat Intense
	4=Intense
	5=Very intense
Q7 Want to close eyes while wearing SCL	0=Never
	1=Rarely
	2=Sometimes
	3=Frequently
	4=Constantly
Q8 Want to remove it while wearing SCL	0=Never
	1=Less than once a week
	2=Weekly
	3=Several times a week
	4=Daily
	5=Several times a day
